# Medicare Payment for Orthopaedic Oncology Procedures Over the Past 20 Years

**DOI:** 10.5435/JAAOSGlobal-D-22-00132

**Published:** 2022-08-05

**Authors:** Jordan R. Pollock, Evan H. Richman, Nicolas P. Kuttner, Joseph C. Brinkman, Nathaniel B. Hinckley, Jack M. Haglin, M. Lane Moore, Sean V. McGarry

**Affiliations:** From the Mayo Clinic Alix School of Medicine, Scottsdale, AZ (Pollock, Haglin, and Moore); the Creighton School of Medicine – Phoenix Regional Campus, Phoenix, AZ (Richman and Kuttner); the Department of Orthopedics, Mayo Clinic Arizona, Phoenix, AZ (Dr. Brinkman and Dr. Hinckley); and the Department of Orthopaedic Surgery & Rehabilitation, University of Nebraska Medical Center, Omaha, NE (Dr. McGarry).

## Abstract

**Background:**

Medicare payment has been examined in a variety of medical and surgical specialties. This study examines Medicare payment in the subspecialty of orthopaedic oncology.

**Methods:**

The Physician Fee Schedule Look-up Tool was used to obtain payment information from 2000 to 2020 for procedures related to orthopaedic oncology billed to Medicare.

**Results:**

For the 38 included orthopaedic oncology procedures, inflation-adjusted Medicare payment decreased an average of 13.6% overall from 2000 to 2020. After adjusting for inflation, the payment for procedures related to spine and pelvis increased by 7.6%, procedures relating to limb salvage increased by 14.6%, procedures associated with the surgical management of complications decreased by 26.9%, and procedures relating to metastatic disease management decreased by 34.8%.

**Conclusion:**

Medicare payment has declined by 13.6% from 2000 to 2020. This variation in Medicare payment represents a difference in valuation of these procedures by the Centers for Medicare and Medicaid Services and could be used to direct healthcare policy.

Medicare insures a large number of patients in the United States, covering those older than 65 years and others with qualifying health conditions.^1,2^ When Medicare was first created in 1965, the median life expectancy in the United States was 70.11 years; in comparison, the life expectancy in 2020 is 78.81 years.^3^ Accordingly, there has been a large increase in the population of Medicare patients in the United States, with the population covered by Medicare increasing from 13.5% of the US population in 2000 to nearly 18.1% in 2019.^4^ The increasing proportion of patients covered by Medicare, and the availability of Medicare payment data, provides an interesting area of economic analysis.

Physicians receive Medicare payment based on the specific medical service provided using a Current Procedural Terminology (CPT) code. Each CPT code is specific to each medical service and is reimbursed based on the valuation of the corresponding relative value units (RVUs). The RVUs for each CPT code are calculated and determined by the Relative Value Update Committee (RUC), which is a coalition of physicians from a variety of specialties.^5^ These payments are also intentionally adjusted by geographic region by Medicare.

Various subspecialties of orthopaedic surgery have reported downward trends in Medicare payment, such as total joint arthroplasty, trauma, hand and wrist, and arthroscopy.^6,7,8,9^ Decreasing Medicare payment is not unique to orthopaedic surgery, with decreases noted among many other specialties including emergency medicine, dermatology, urology, and neurosurgery.^10,11,12,13^ Current literature lacks an updated, long-term Medicare payment study in the field of musculoskeletal oncology. This study further defines the trends of inflation-adjusted Medicare payment in the field of musculoskeletal oncology.

## Methods

The Medicare database used in this study is publicly available. Accordingly, this study did not require an institutional review board approval. Trends in Medicare payment have been examined for many subspecialties of orthopaedic surgery.^7,9-11,14-16^ Accordingly, the methods of this study were adapted from these previous studies to facilitate comparison between these studies and our study.

### Data Extraction

A list of procedures and their corresponding CPT codes representing orthopaedic oncology was obtained from the orthopaedic oncology fellowship Accreditation Council for Graduate Medical Education (ACGME) case log guidelines. These guidelines contain a categorized list of procedures pertaining to orthopaedic oncology.^17^ Only procedures common to 2000 and 2020 were included in this analysis. Accordingly, nine codes that did not exist in the year 2000 were excluded.

We obtained payment information from the CMS website using the Physician Fee Schedule Look-Up Tool for each CPT code in this study.^18^ Payment information was averaged for each geographic Medicare Administrative Contractor locality from 2000 to 2020 to obtain a yearly national average payment for each code. To adjust for inflation between 2000 and 2020, the national average payment information for each code was multiplied by the inflation multiplier for each year, which was calculated using the change in Consumer Price Index from the US Department of Labor, Bureau of Labor Statistics website.^19^ As such, inflation-adjusted amounts were adjusted to the worth of a US dollar as of January 1, 2021.

### Statistical Analysis

Payment averages were compared by calculating the percentage change from 2000 to 2020 after adjusting for inflation. These results were analyzed using a two-tailed Student *t*-test comparison of means with alpha <0.05. We also conducted these same analyses for each category of musculoskeletal oncology procedures according to their designation in the ACGME case log. These categories were spine and pelvis, limb salvage, surgical management of complications, and metastatic management. A compound annual growth rate was also included for each code, along with an r-squared value. The statistical analysis conducted in this study was calculated using Microsoft Excel for Office 365 (Microsoft).

## Results

Our analysis included 38 CPT codes after excluding nine codes that did not exist in the year 2000 (Supplemental Table 1, http://links.lww.com/JG9/A227). The mean Medicare payment for the included 38 CPT codes after adjusting 2000 dollars to 2020 dollars for inflation decreased from $1,738 on average in 2000 to $1,452 on average in 2020 (13.6% decrease). The median inflation-adjusted payment of all codes from 2000 to 2020 decreased by 25.3%, and the payment change for each code from 2000 to 2020 ranged between a 51.4% increase and a 44.4% decrease (Table 1).

**Table 1:** All 38 Included Procedures and Their Inflation-adjusted Payment in 2000 and 2020, Sorted by Largest to Smallest Percentage Change

Procedure	CPT Code	2000 Reimbursement	2020 Reimbursement	Percentage change 00–20	CAGR	R sq
Open treatment of proximal humeral (surgical or anatomical neck) fracture, includes internal fixation, when performed, includes repair of tuberosity(s), when performed; with proximal humeral prosthetic replacement	23616	2401	1307	−44.4%	−3.2%	0.84
Arthroplasty, knee, condyle and plateau; medial AND lateral compartments with or without patella resurfacing (total knee arthroplasty)	27447	2486	1430	−41.3%	−2.9%	0.92
Prophylactic treatment (nailing, pinning, plating or wiring) with or without methylmethacrylate, femoral neck and proximal femur	27187	1623	1052	−38.9%	−2.3%	0.81
Open treatment of intertrochanteric, peritrochanteric, or subtrochanteric femoral fracture; with intramedullary implant, with or without interlocking screws and/or cerclage	27245	2143	1298	−38.1%	−2.6%	0.88
Arthroplasty, acetabular and proximal femoral prosthetic replacement (total hip arthroplasty), with or without autograft or allograft	27130	2349	1432	−37.8%	−2.6%	0.85
Repair, nonunion or malunion, femur, distal to head and neck; with iliac or other autogenous bone graft (includes obtaining graft)	27472	2111	1335	−35.5%	−2.4%	0.80
Prophylactic treatment (nailing, pinning, plating, or wiring) with or without methylmethacrylate, femur	27495	1873	1192	−35.1%	−2.4%	0.79
Open treatment of femoral supracondylar or transcondylar fracture without intercondylar extension, includes internal fixation, when performed	27511	1624	1055	−33.9%	−2.2%	0.85
Open treatment of femoral supracondylar or transcondylar fracture with intercondylar extension, includes internal fixation, when performed	27513	1980	1311	−32.5%	−2.1%	0.89
Prophylactic treatment (nailing, pinning, plating or wiring) with or without methylmethacrylate, tibia	27745	1192	804	−31.7%	−2.0%	0.77
Prophylactic treatment (nailing, pinning, plating or wiring), with or without methylmethacrylate, humeral shaft	24498	1364	915	−31.5%	−2.1%	0.80
Prophylactic treatment (nailing, pinning, plating or wiring) with or without methylmethacrylate; proximal humerus	23491	1586	1073	−30.9%	−2.0%	0.82
Hemiarthroplasty, hip, partial (eg, femoral stem prosthesis, bipolar arthroplasty)	27125	1739	1196	−29.7%	−1.9%	0.72
Removal of prosthesis, including total knee prosthesis, methylmethacrylate with or without insertion of spacer, knee	27488	1823	1268	−29.0%	−1.9%	0.78
Removal of hip prosthesis; complicated, including total hip prosthesis, methylmethacrylate with or without insertion of spacer	27091	2406	1686	−28.5%	−1.9%	0.77
Repair of nonunion or malunion, humerus; with iliac or other autograft (includes obtaining graft)	24435	1598	1136	−27.3%	−1.8%	0.75
Open treatment of humeral shaft fracture with plate/screws, with or without cerclage	24515	1290	929	−26.7%	−1.7%	0.81
Treatment of intertrochanteric, peritrochanteric, or subtrochanteric femoral fracture; with plate/screw type implant, with or without cerclage	27244	1808	1299	−26.6%	−1.7%	0.72
Muscle, myocutaneous, or fasciocutaneous flap; upper extremity	15736	1775	1293	−25.5%	−1.7%	0.72
Open treatment of femoral shaft fracture, with or without external fixation, with insertion of intramedullary implant, with or without cerclage and/or locking screws	27506	1928	1413	−25.2%	−1.6%	0.83
Repair of nonunion or malunion, tibia; with iliac or other autograft (includes obtaining graft)	27724	1778	1330	−23.4%	−1.5%	0.80
Muscle, myocutaneous, or fasciocutaneous flap; lower extremity	15738	1793	1370	−21.4%	−1.4%	0.79
Radical resection of tumor; talus or calcaneus	27647	1323	1077	−16.7%	−1.1%	0.14
Partial excision of posterior vertebral component (eg, spinous process, lamina or facet) for intrinsic bony lesion, single vertebral segment; thoracic	22101	1092	915	−15.4%	−0.9%	0.42
Partial excision of vertebral body, for intrinsic bony lesion, without decompression of spinal cord or nerve root(s), single vertebral segment; thoracic	22112	1380	1170	−12.4%	−0.9%	0.34
Partial excision of posterior vertebral component (eg, spinous process, lamina or facet) for intrinsic bony lesion, single vertebral segment; lumbar	22102	989	864	−11.3%	−0.7%	0.80
Partial excision of vertebral body, for intrinsic bony lesion, without decompression of spinal cord or nerve root(s), single vertebral segment; lumbar	22114	1345	1170	−10.1%	−0.7%	0.52
Radical resection of tumor, shaft or distal humerus	24150	1603	1637	4.6%	0.1%	0.21
Radical resection of tumor; fibula	27646	1537	1626	8.3%	0.3%	0.15
Radical resection of tumor, radius or ulna	25170	1376	1556	15.8%	0.6%	0.15
Radical resection of tumor; tibia	27645	1650	1872	16.3%	0.7%	0.26
Radical resection of tumor; ilium, including acetabulum, both pubic rami, or ischium and acetabulum	27076	2356	2673	16.3%	0.7%	0.39
Radical resection of tumor; wing of ilium, 1 pubic or ischial ramus or symphysis pubis	27075	1882	2209	20.3%	0.8%	0.17
Radical resection of tumor; innominate bone, total	27077	2503	2983	22.2%	0.9%	0.01
Radical resection of tumor, femur or knee	27365	1801	2177	23.6%	1.0%	0.50
Radical resection of tumor, proximal humerus	23220	1656	2058	26.9%	1.1%	0.47
Radical resection of tumor; scapula	23210	1388	1872	38.3%	1.6%	0.50
Radical resection of tumor; ischial tuberosity and greater trochanter of femur	27078	1475	2178	51.4%	2.1%	0.56
Median		1698	1309	−25.3%	−1.6%	0.76
Mean		1738	1452	−13.6%	−1.1%	0.61

CPT = Current Procedural Terminology, CAGR = Compound Annual Growth Rate

The CPT codes and descriptions in this table were adapted from the orthopaedic surgery oncology fellowship ACGME case logs.^17^

**Figure 1:**
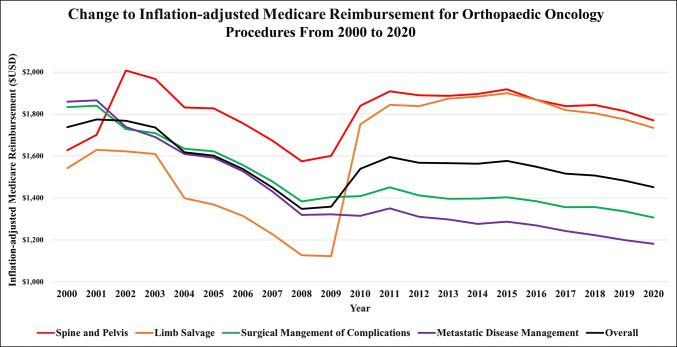
Graph showing the change in payment for orthopaedic oncology procedures from 2000 to 2020.

The categories of spine and pelvis and limb salvage experienced increases in inflation-adjusted Medicare payment of 7.6% and 14.6%, respectively. Inflation-adjusted Medicare payment decreased for surgical management of complications (−26.9%) and metastatic management (−34.8%) between the years of 2000 and 2020 (Figure 1 and Table 2).

**Table 2:** Summary of Differences in Inflation-adjusted Medicare Payment Between Orthopaedic Oncology Procedures

Orthopaedic Oncology Procedures	No. of CPT Codes	Average Inflation-adjusted Medicare Payment 2000	Average Inflation-adjusted Medicare Payment 2020	Percentage Change
Spine and pelvis	8	1628	1770	7.6%
Limb salvage	8	1542	1734	14.6%
Surgical management of complications	9	1834	1307	−26.9%
Metastatic management	13	1859	1182	−34.8%
Overall	38	1738	1452	−13.6%

CPT = Current Procedural Terminology

Inflation-adjusted payment by Medicare for eight spine and pelvis procedures increased from $1,628 in 2000 to $1,770 in 2020 (7.6% increase). The largest decrease was seen for CPT code 22101 (partial excision of posterior vertebral component for intrinsic bony lesion, single vertebral segment; thoracic), which experienced a 15.4% decrease, whereas the largest increase in payment was seen for CPT code 27078 (radical resection of tumor; ischial tuberosity and greater trochanter of femur) at 51.4% (Table 3).

**Table 3:** Orthopaedic Oncology Procedures Related to Spine and Pelvis Inflation-adjusted Medicare Payment From 2000 to 2020

Description	CPT Code	2000	2020	Percentage change	CAGR	R Sq
Partial excision of posterior vertebral component (eg, spinous process, lamina or facet) for intrinsic bony lesion, single vertebral segment; thoracic	22101	1092	915	−15.4%	−0.9%	0.42
Partial excision of vertebral body, for intrinsic bony lesion, without decompression of spinal cord or nerve root(s), single vertebral segment; thoracic	22112	1380	1170	−12.4%	−0.9%	0.34
Partial excision of posterior vertebral component (eg, spinous process, lamina or facet) for intrinsic bony lesion, single vertebral segment; lumbar	22102	989	864	−11.3%	−0.7%	0.80
Partial excision of vertebral body, for intrinsic bony lesion, without decompression of spinal cord or nerve root(s), single vertebral segment; lumbar	22114	1345	1170	−10.1%	−0.7%	0.52
Radical resection of tumor; ilium, including acetabulum, both pubic rami, or ischium and acetabulum	27076	2356	2673	16.3%	0.7%	0.39
Radical resection of tumor; wing of ilium, 1 pubic or ischial ramus or symphysis pubis	27075	1882	2209	20.3%	0.8%	0.17
Radical resection of tumor; innominate bone, total	27077	2503	2983	22.2%	0.9%	0.01
Radical resection of tumor; ischial tuberosity and greater trochanter of femur	27078	1475	2178	51.4%	2.1%	0.56
Median		1428	1674	3.1%	0.0%	0.41
Mean		1628	1770	7.6%	0.2%	0.40

CPT = Current Procedural Terminology

The CPT codes and descriptions in this table were adapted from the orthopaedic surgery oncology fellowship ACGME case logs.^17^

Inflation-adjusted Medicare payment for the eight limb salvage procedures increased from $1,542 in 2000 to $1,734 in 2020 (14.6% increase). All eight procedures in this category increased in inflation-adjusted payment by Medicare from 2000 to 2020. The smallest increase was seen for CPT code 27647 (radical resection of tumor; talus or calcaneus), which experienced a 4.6% increase, and the largest increase was seen for CPT code 23210 (radical resection of tumor; scapula) at 38.3% (Table 4).

**Table 4:** Orthopaedic Oncology Procedures Related to Limb Salvage Inflation-adjusted Medicare Payment From 2000 to 2020

Description	CPT Code	2000	2020	Percentage change	CAGR	R Sq
Radical resection of tumor; talus or calcaneus	27647	1323	1077	−16.7%	−1.1%	0.14
Radical resection of tumor, shaft or distal humerus	24150	1603	1637	4.6%	0.1%	0.21
Radical resection of tumor; fibula	27646	1537	1626	8.3%	0.3%	0.15
Radical resection of tumor, radius or ulna	25170	1376	1556	15.8%	0.6%	0.15
Radical resection of tumor; tibia	27645	1650	1872	16.3%	0.7%	0.26
Radical resection of tumor, femur or knee	27365	1801	2177	23.6%	1.0%	0.50
Radical resection of tumor, proximal humerus	23220	1656	2058	26.9%	1.1%	0.47
Radical resection of tumor; scapula	23210	1388	1872	38.3%	1.6%	0.50
Median		1570	1754	16.1%	0.7%	0.24
Mean		1542	1734	14.6%	0.5%	0.30

CPT = Current Procedural Terminology

The CPT codes and descriptions in this table were adapted from the orthopaedic surgery oncology fellowship ACGME case logs.^17^

Inflation-adjusted Medicare payment for surgical complications decreased from $1,834 in 2000 to $1,307 in 2020 (−26.9% decrease). The largest decrease was seen for CPT code 27472 (repair, nonunion or malunion, femur, distal to head and neck; with iliac or other autogenous bone graft, including obtaining graft), which experienced a −35.5% decrease, and the smallest decrease was seen for CPT code 15738 (muscle, myocutaneous, or fasciocutaneous flap; lower extremity) at −21.4% (Table 5).

**Table 5:** Orthopaedic Oncology Procedures Related to Management of Surgical Complications Inflation-adjusted Medicare Payment From 2000 to 2020

Description	CPT code	2000	2020	Percentage change	CAGR	R Sq
Repair, nonunion or malunion, femur, distal to head and neck; with iliac or other autogenous bone graft (includes obtaining graft)	27472	2111	1335	−35.5%	−2.4%	0.80
Removal of prosthesis, including total knee prosthesis, methylmethacrylate with or without insertion of spacer, knee	27488	1823	1268	−29.0%	−1.9%	0.78
Removal of hip prosthesis; complicated, including total hip prosthesis, methylmethacrylate with or without insertion of spacer	27091	2406	1686	−28.5%	−1.9%	0.77
Repair of nonunion or malunion, humerus; with iliac or other autograft (includes obtaining graft)	24435	1598	1136	−27.3%	−1.8%	0.75
Open treatment of humeral shaft fracture with plate/screws, with or without cerclage	24515	1290	929	−26.7%	−1.7%	0.81
Muscle, myocutaneous, or fasciocutaneous flap; upper extremity	15736	1775	1293	−25.5%	−1.7%	0.72
Open treatment of femoral shaft fracture, with or without external fixation, with insertion of intramedullary implant, with or without cerclage and/or locking screws	27506	1928	1413	−25.2%	−1.6%	0.83
Repair of nonunion or malunion, tibia; with iliac or other autograft (includes obtaining graft)	27724	1778	1330	−23.4%	−1.5%	0.80
Muscle, myocutaneous, or fasciocutaneous flap; lower extremity	15738	1793	1370	−21.4%	−1.4%	0.79
Median		1793	1330	−26.7%	−1.7%	0.79
Mean		1834	1307	−26.9%	−1.8%	0.78

CPT = Current Procedural Terminology

The CPT codes and descriptions in this table were adapted from the orthopaedic surgery oncology fellowship ACGME case logs.^17^

Inflation-adjusted Medicare payment for metastatic management decreased from $1,859 in 2000 to $1,182 in 2020 (−34.8% decrease). The largest decrease was seen for CPT code 23616 (open treatment of proximal humeral fracture), which experienced a −41.3% decrease, and the smallest decrease was seen for CPT code 27244 (treatment of intertrochanteric, peritrochanteric, or subtrochanteric femoral fracture; with plate/screw type implant, with or without cerclage) at −26.6% (Table 6).

**Table 6:** Orthopaedic Oncology Procedures Related to Metastatic Management Inflation-adjusted Medicare Payment From 2000 to 2020

Description	CPT code	2000	2020	Percentage change	CAGR	R Sq
Open treatment of proximal humeral (surgical or anatomical neck) fracture, includes internal fixation, when performed, includes repair of tuberosity(s), when performed; with proximal humeral prosthetic replacement	23616	2401	1307	−44.4%	−3.2%	0.84
Arthroplasty, knee, condyle and plateau; medial AND lateral compartments with or without patella resurfacing (total knee arthroplasty)	27447	2486	1430	−41.3%	−2.9%	0.92
Prophylactic treatment (nailing, pinning, plating or wiring) with or without methylmethacrylate, femoral neck and proximal femur	27187	1623	1052	−38.9%	−2.3%	0.81
Open treatment of intertrochanteric, peritrochanteric, or subtrochanteric femoral fracture; with intramedullary implant, with or without interlocking screws and/or cerclage	27245	2143	1298	−38.1%	−2.6%	0.88
Arthroplasty, acetabular and proximal femoral prosthetic replacement (total hip arthroplasty), with or without autograft or allograft	27130	2349	1432	−37.8%	−2.6%	0.85
Prophylactic treatment (nailing, pinning, plating, or wiring) with or without methylmethacrylate, femur	27495	1873	1192	−35.1%	−2.4%	0.79
Open treatment of femoral supracondylar or transcondylar fracture without intercondylar extension, includes internal fixation, when performed	27511	1624	1055	−33.9%	−2.2%	0.85
Open treatment of femoral supracondylar or transcondylar fracture with intercondylar extension, includes internal fixation, when performed	27513	1980	1311	−32.5%	−2.1%	0.89
Prophylactic treatment (nailing, pinning, plating or wiring) with or without methylmethacrylate, tibia	27745	1192	804	−31.7%	−2.0%	0.77
Prophylactic treatment (nailing, pinning, plating or wiring), with or without methylmethacrylate, humeral shaft	24498	1364	915	−31.5%	−2.1%	0.80
Prophylactic treatment (nailing, pinning, plating or wiring) with or without methylmethacrylate; proximal humerus	23491	1586	1073	−30.9%	−2.0%	0.82
Hemiarthroplasty, hip, partial (eg, femoral stem prosthesis, bipolar arthroplasty)	27125	1739	1196	−29.7%	−1.9%	0.72
Treatment of intertrochanteric, peritrochanteric, or subtrochanteric femoral fracture; with plate/screw type implant, with or without cerclage	27244	1808	1299	−26.6%	−1.7%	0.72
Median		1808	1196	−33.9%	−2.2%	0.82
Mean		1859	1182	−34.8%	−2.3%	0.82

CPT = Current Procedural Terminology

The CPT codes and descriptions in this table were adapted from the orthopaedic surgery oncology fellowship ACGME case logs.^17^

## Discussion

In musculoskeletal oncology, inflation-adjusted Medicare payment has decreased between the years of 2000 to 2020, with a 13.6% mean decrease overall, with a maximum decrease in payment of 44.4% for open treatment of the proximal humerus and a maximum increase in payment of 51.4% for the radical resection of tumor from the ischial tuberosity and greater trochanter of the femur. The orthopaedic oncology procedures associated with increases in inflation-adjusted Medicare payment are spine and pelvis and limb salvage, whereas the procedures with the largest decrease in payment were related to surgical complications and metastatic management. Orthopaedic oncology has experienced a similar decrease in comparison with other subspecialties of orthopaedic surgery. For example, Medicare payment after adjusting for inflation decreased 39% for knee arthroplasty and hip arthroplasty from 2000 to 2019, on average.^7^ Inflation-adjusted Medicare payment decreased for shoulder and elbow by 27% from 2002 to 2018.^20^ Inflation-adjusted Medicare payment for arthroscopy procedures decreased from 2000 to 2019 by 30% for commonly performed arthroscopy procedures.^15^

Our analysis also revealed that categories of orthopaedic oncology surgery are affected to different degrees by decreases in Medicare payment. For example, spine, pelvis, and limb salvage procedures increased in payment by Medicare from 2000 to 2020, whereas surgical complication management and metastatic management decreased in payment. It has been reported that the RVUs may not accurately reflect surgeon work, and these findings can help contextualize and quantify payments for comparison.^21^

Medicare payment is markedly less than commercial payors, but this difference varies by procedure. A study examining payment trends for shoulder arthroscopy found that payment from private payors was more than double of Medicare payment, while private payment for shoulder arthroscopy: capsulorrhaphy was only 16% more than Medicare payment.^22^ Another study found that both commercial and Medicare payment for an orthopaedic procedure has decreased from 2010 to 2018, with payments by Medicare decreasing 1.5 times faster than commercial payments, on average.^23^ Payment trends of large, commercial payors warrants future analysis to further define these trends.

Healthcare policy changes over the past two decades have likely affected the payment trends seen in our study, for example, the Balanced Budget Act of 1997 and the Medicare Access and CHIP Reauthorization Act of 2015. Although these policies have likely influenced the payment trends seen in our study, annual adjustments to CPT code pricing are also directly responsible for the changes seen in our study. CPT code valuations are updated on an annual basis by the RUC, a committee of volunteer physicians from different specialties.^24^ This committee currently includes one orthopaedic surgeon.^24^ To determine valuations and payment changes, RUC physicians adjust the valuation of CPT codes by adjusting the associated RVUs. These changes can occur through modification of one, two, or three RVU components of differing weights, consisting of work RVU, practice expense RVU, and malpractice RVU. Payment for medical services is also affected by geographic adjustments named the work geographic pricing cost index (GPCI), practice expense GPCI, and malpractice GPCI. RVUs and GPCIs are multiplied together and assigned weights. Subsequently, the resulting value is multiplied by the corresponding year's conversion factor to determine physician payment. Notably, the conversion factor affects all CPT codes in medicine equally and has decreased slightly throughout 2000 to 2020, being $36.61 in 2000 and $36.09 in 2020.^25^

A few limitations should be mentioned for this study. The codes included in our study do not represent all the possible procedures and services performed by orthopaedic oncologists. In addition, this study uses data from a single payor, and as such, the trends observed in our study may not represent payment by third party payors. Additional study is needed to elucidate trends in payment amount from third party payors from 2000 to 2020.

## Conclusion

Medicare payment for orthopaedic oncology has declined 13.6% from 2000 to 2020. Although the payment for spine and pelvic procedures increased by 7.6%, payment for procedures relating to metastatic disease management decreased by 34.8%. This variation in Medicare payment represents a difference in valuation of these procedures by the CMS and could be used to direct healthcare policy. An understanding of these trends is essential because hospitals, physicians, and policy makers can use this information to address declining payment while also continuing to ensure equitable access to care for Medicare patients.
